# 2-Methyl-3-(1*H*-pyrazol-3-yl)imidazo[1,2-*a*]pyrimidine

**DOI:** 10.1107/S1600536812024166

**Published:** 2012-06-02

**Authors:** Guo-Rui Gao, Wen-Hu Duan

**Affiliations:** a School of Pharmacy, East China University of Science and Technology, Shanghai 200237, People’s Republic of China; b Department of Medicinal Chemistry, Shanghai Institute of Materia Medica, Chinese Academy of Sciences, Shanghai 201203, People’s Republic of China

## Abstract

In the title compound, C_10_H_9_N_5_, the fused 2-methyl­imidazo[1,2-*a*]pyrimidine ring system is approximately planar [dihedral angle of 1.14 (9)° between the two fused rings] and the 1*H*-pyrazole ring is rotated by 28.16 (11)° out of that plane. In the crystal, the mol­ecules are linked into linear chains along the [100] direction by classical inter­molecular N—H⋯N hydrogen bonds.

## Related literature

For the medical properties of imidazo[1,2-*a*]pyrimidine derivatives, see: An *et al.* (2009 ▶); Kim *et al.* (2011 ▶); Linton *et al.* (2011 ▶). For related structures, see: Yang *et al.* (2008 ▶); Anaflous *et al.* (2004 ▶).
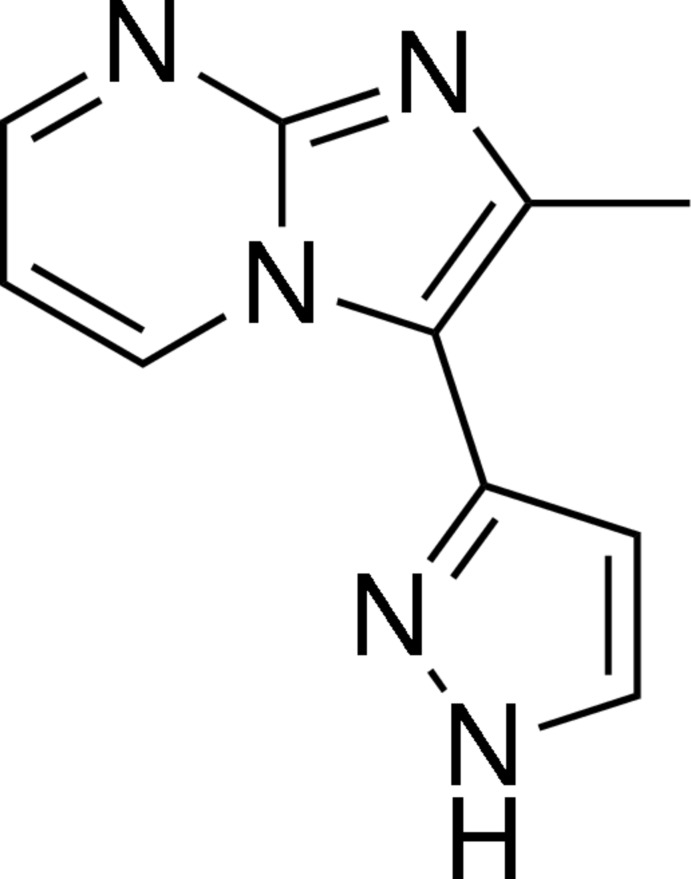


## Experimental

### 

#### Crystal data

C_10_H_9_N_5_
*M* *_r_* = 199.22
Monoclinic, 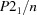

*a* = 8.962 (2) Å
*b* = 8.851 (2) Å
*c* = 12.481 (3) Å
β = 101.751 (3)°
*V* = 969.3 (4) Å^3^
*Z* = 4
Mo *K*α radiation
μ = 0.09 mm^−1^
*T* = 173 K
0.50 × 0.10 × 0.08 mm

#### Data collection

Bruker SMART APEX CCD diffractometer
Absorption correction: multi-scan (*SADABS*; Bruker, 2000 ▶) *T*_min_ = 0.956, *T*_max_ = 0.993
5121 measured reflections
1907 independent reflections
1552 reflections with *I* > 2σ(*I*)
*R*_int_ = 0.024

#### Refinement

*R*[*F* ^2^ > 2σ(*F* ^2^)] = 0.051
*wR*(*F* ^2^) = 0.128
*S* = 1.09
1907 reflections
152 parameters
H atoms treated by a mixture of independent and constrained refinement
Δρ_max_ = 0.20 e Å^−3^
Δρ_min_ = −0.14 e Å^−3^

### 

Data collection: *SMART* (Bruker, 2000 ▶); cell refinement: *SMART*; data reduction: *SAINT* (Bruker, 2000 ▶); program(s) used to solve structure: *SHELXS97* (Sheldrick, 2008 ▶); program(s) used to refine structure: *SHELXL97* (Sheldrick, 2008 ▶); molecular graphics: *SHELXTL* (Sheldrick, 2008 ▶); software used to prepare material for publication: *SHELXTL*.

## Supplementary Material

Crystal structure: contains datablock(s) I, global. DOI: 10.1107/S1600536812024166/rk2359sup1.cif

Structure factors: contains datablock(s) I. DOI: 10.1107/S1600536812024166/rk2359Isup2.hkl

Supplementary material file. DOI: 10.1107/S1600536812024166/rk2359Isup3.cml

Additional supplementary materials:  crystallographic information; 3D view; checkCIF report

## Figures and Tables

**Table 1:** Hydrogen-bond geometry (Å, °)

*D*—H⋯*A*	*D*—H	H⋯*A*	*D*⋯*A*	*D*—H⋯*A*
N12—H30*A*⋯N6^i^	0.89 (2)	2.08 (2)	2.966 (2)	175 (2)

Symmetry code: (i) 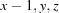.

**Fig. 1.**
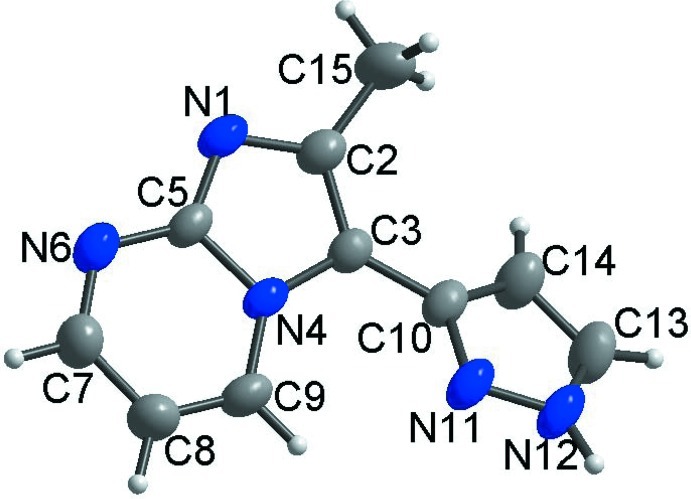
Molecular structure of the title compound, with the atom-numbering scheme. Displacement ellipsoids are drawn at the 30% probability level. H atoms are presented as a small spheres of arbitrary radius.

**Fig. 2.**
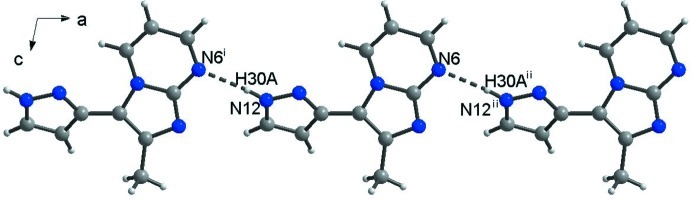
The one-dimensional chain structure extending along [100], with hydrogen bonds shown as dashed lines. Symmetry codes: (i) x - 1, y, z; (ii) x + 1, y, z.

## supplementary crystallographic information

### Comment

Imidazo[1,2-*a*]pyrimidine derivatives have been used for treatment of cancers (An *et al.*, 2009; Kim *et al.*, 2011; Linton *et al.*, 2011), and some analogues were reported (Anaflous *et al.*, 2004; Yang *et al.*, 2008). The title compound was synthesized by the reaction of 1-(2-methylimidazo[1,2-*a*]pyrimidin-3-yl)ethanone with 1,1-dimethoxy-*N*,*N*-dimethylmethanamine, and its crystal structure was confirmed by single crystal X-ray analysis (Fig. 1). In this compound, the fused 2-methylimidazo[1,2-*a*]pyrimidine ring system are approximately coplanar with a tiny dihedral angle being 1.14 (9)°, but the 1*H*-pyrazole ring is rotated 28.16 (11)° out of the the fused 2-methylimidazo[1,2-*a*]pyrimidine ring system plane. Further analysis showed that each molecule is associated with two neighbors to generate an infinite one-dimensional chain extending along the [1 0 0] direction with the formation classical intermolecular N12—H30A···N6^i^ hydrogen bond (Fig. 2). Symmetry code: (i) *x* - 1, *y*, *z*. The relevant parameters are listed in Table 1.

### Experimental

A mixture of 1-(2-methylimidazo[1,2-*a*]pyrimidin-3-yl)ethanone (500 mg, 2.86 mmol) and 1,1-dimethoxy-*N*,*N*-dimethylmethanamine (3.8 ml, 28.6 mmol) was heated at 373 K for 6 h. After cooling to room temperature, EtOH (10 ml) and hydrazine hydrate (0.28 ml, 5.72 mmol) were added and the reaction mixture was refluxed for 3 h. The mixture was diluted with water and extracted with CH_2_Cl_2_. The organic layer was dried over MgSO_4_ and evaporated. The crude product was chromatographed on silica gel eluting with DCM–MeOH (10/1) to give the title compound as a pale-yellow solid (300 mg, yield: 52%).

### Refinement

H atoms attached to NH group and methyl C atoms were located in a difference Fourier map and refined isotropically. The other H atoms attached to C atoms were positioned with idealized geometry (C—H = 0.95Å) using a riding model with *U*_iso_(H) = 1.2*U*_eq_(C).

### Crystal data

**Table:**

C_10_H_9_N_5_	*F*(000) = 416
*M**_r_* = 199.22	*D*_x_ = 1.365 Mg m^−^^3^
Monoclinic, *P*2_1_/*n*	Mo *K*α radiation, λ = 0.71073 Å
Hall symbol: -P 2yn	Cell parameters from 1511 reflections
*a* = 8.962 (2) Å	θ = 2.6–24.0°
*b* = 8.851 (2) Å	µ = 0.09 mm^−^^1^
*c* = 12.481 (3) Å	*T* = 173 K
β = 101.751 (3)°	Bar, pale yellow
*V* = 969.3 (4) Å^3^	0.50 × 0.10 × 0.08 mm
*Z* = 4	

### Data collection

**Table:**

Bruker SMART APEX CCD diffractometer	1907 independent reflections
Radiation source: fine-focus sealed tube	1552 reflections with *I* > 2σ(*I*)
Graphite monochromator	*R*_int_ = 0.024
φ and ω scans	θ_max_ = 26.0°, θ_min_ = 2.6°
Absorption correction: multi-scan (*SADABS*; Bruker, 2000)	*h* = −10→11
*T*_min_ = 0.956, *T*_max_ = 0.993	*k* = −10→10
5121 measured reflections	*l* = −15→8

### Refinement

**Table:**

Refinement on *F*^2^	Primary atom site location: structure-invariant direct methods
Least-squares matrix: full	Secondary atom site location: difference Fourier map
*R*[*F*^2^ > 2σ(*F*^2^)] = 0.051	Hydrogen site location: inferred from neighbouring sites
*wR*(*F*^2^) = 0.128	H atoms treated by a mixture of independent and constrained refinement
*S* = 1.09	*w* = 1/[σ^2^(*F*_o_^2^) + (0.0572*P*)^2^ + 0.2361*P*] where *P* = (*F*_o_^2^ + 2*F*_c_^2^)/3
1907 reflections	(Δ/σ)_max_ < 0.001
152 parameters	Δρ_max_ = 0.20 e Å^−^^3^
0 restraints	Δρ_min_ = −0.14 e Å^−^^3^

### Special details

**Table:**

Geometry. All s.u.'s (except the s.u. in the dihedral angle between two l.s. planes) are estimated using the full covariance matrix. The cell s.u.'s are taken into account individually in the estimation of s.u.'s in distances, angles and torsion angles; correlations between s.u.'s in cell parameters are only used when they are defined by crystal symmetry. An approximate (isotropic) treatment of cell s.u.'s is used for estimating s.u.'s involving l.s. planes.
Refinement. Refinement of *F*^2^ against ALL reflections. The weighted *R*-factor *wR* and goodness of fit *S* are based on *F*^2^, conventional *R*-factors *R* are based on *F*, with *F* set to zero for negative *F*^2^. The threshold expression of *F*^2^ > σ(*F*^2^) is used only for calculating *R*-factors(gt) *etc*. and is not relevant to the choice of reflections for refinement. *R*-factors based on *F*^2^ are statistically about twice as large as those based on *F*, and *R*-factors based on ALL data will be even larger.

### Fractional atomic coordinates and isotropic or equivalent isotropic displacement parameters (Å^2^)

**Table:**

	*x*	*y*	*z*	*U*_iso_*/*U*_eq_	
N4	0.44313 (15)	0.15343 (16)	0.08173 (12)	0.0371 (4)	
N1	0.65249 (16)	0.11695 (18)	0.21150 (13)	0.0455 (4)	
N6	0.68599 (17)	0.2102 (2)	0.03899 (14)	0.0495 (4)	
C3	0.39442 (19)	0.0960 (2)	0.17190 (15)	0.0376 (4)	
N12	−0.00496 (19)	0.0927 (2)	0.13228 (16)	0.0531 (5)	
C5	0.60176 (19)	0.1626 (2)	0.11018 (15)	0.0401 (4)	
C9	0.3688 (2)	0.1977 (2)	−0.01970 (16)	0.0475 (5)	
H9A	0.2609	0.1930	−0.0399	0.057*	
C10	0.23463 (19)	0.0676 (2)	0.17285 (15)	0.0399 (4)	
N11	0.12533 (17)	0.14869 (19)	0.10998 (14)	0.0492 (5)	
C2	0.5255 (2)	0.0765 (2)	0.24983 (15)	0.0431 (5)	
C13	0.0186 (2)	−0.0174 (2)	0.2049 (2)	0.0565 (6)	
H13A	−0.0573	−0.0719	0.2319	0.068*	
C8	0.4521 (2)	0.2482 (3)	−0.09045 (17)	0.0560 (6)	
H8A	0.4038	0.2812	−0.1615	0.067*	
C14	0.1724 (2)	−0.0382 (2)	0.23391 (19)	0.0536 (5)	
H14A	0.2255	−0.1094	0.2847	0.064*	
C7	0.6118 (2)	0.2516 (3)	−0.05784 (18)	0.0560 (6)	
H7A	0.6692	0.2859	−0.1092	0.067*	
C15	0.5385 (3)	0.0238 (4)	0.3649 (2)	0.0648 (7)	
H30A	−0.095 (3)	0.129 (3)	0.1009 (18)	0.061 (7)*	
H15C	0.478 (4)	−0.057 (4)	0.372 (3)	0.130 (13)*	
H15B	0.639 (4)	−0.005 (4)	0.396 (3)	0.119 (12)*	
H15A	0.512 (5)	0.106 (6)	0.410 (4)	0.184 (19)*	

### Atomic displacement parameters (Å^2^)

**Table:**

	*U*^11^	*U*^22^	*U*^33^	*U*^12^	*U*^13^	*U*^23^
N4	0.0254 (7)	0.0391 (8)	0.0440 (9)	0.0020 (6)	0.0003 (6)	−0.0010 (6)
N1	0.0289 (8)	0.0517 (10)	0.0521 (10)	−0.0001 (7)	−0.0008 (7)	0.0043 (7)
N6	0.0318 (9)	0.0591 (10)	0.0575 (11)	−0.0004 (7)	0.0089 (7)	0.0040 (8)
C3	0.0303 (9)	0.0368 (9)	0.0446 (10)	0.0011 (7)	0.0051 (7)	−0.0007 (8)
N12	0.0259 (9)	0.0593 (11)	0.0735 (12)	0.0001 (7)	0.0086 (8)	0.0012 (9)
C5	0.0259 (9)	0.0429 (10)	0.0492 (11)	0.0010 (7)	0.0026 (7)	−0.0013 (8)
C9	0.0325 (10)	0.0550 (12)	0.0504 (11)	0.0036 (8)	−0.0024 (8)	0.0025 (9)
C10	0.0305 (9)	0.0376 (10)	0.0506 (11)	0.0012 (7)	0.0060 (8)	−0.0044 (8)
N11	0.0265 (8)	0.0531 (10)	0.0666 (11)	0.0001 (7)	0.0063 (7)	0.0046 (8)
C2	0.0344 (10)	0.0436 (11)	0.0485 (11)	−0.0003 (8)	0.0019 (8)	0.0015 (8)
C13	0.0412 (11)	0.0518 (12)	0.0803 (16)	−0.0067 (9)	0.0212 (10)	0.0038 (11)
C8	0.0467 (12)	0.0715 (14)	0.0474 (12)	0.0043 (10)	0.0040 (9)	0.0095 (10)
C14	0.0393 (11)	0.0500 (12)	0.0720 (14)	0.0008 (9)	0.0124 (10)	0.0112 (10)
C7	0.0455 (12)	0.0683 (14)	0.0565 (13)	−0.0008 (10)	0.0156 (10)	0.0093 (11)
C15	0.0530 (15)	0.0809 (18)	0.0544 (14)	−0.0088 (13)	−0.0037 (11)	0.0185 (13)

### Geometric parameters (Å, º)

**Table:**

N4—C9	1.362 (2)	C9—H9A	0.9500
N4—C3	1.384 (2)	C10—N11	1.333 (2)
N4—C5	1.396 (2)	C10—C14	1.394 (3)
N1—C5	1.317 (2)	C2—C15	1.492 (3)
N1—C2	1.369 (2)	C13—C14	1.364 (3)
N6—C7	1.307 (3)	C13—H13A	0.9500
N6—C5	1.345 (2)	C8—C7	1.406 (3)
C3—C2	1.375 (2)	C8—H8A	0.9500
C3—C10	1.456 (2)	C14—H14A	0.9500
N12—C13	1.318 (3)	C7—H7A	0.9500
N12—N11	1.349 (2)	C15—H15C	0.92 (4)
N12—H30A	0.89 (2)	C15—H15B	0.94 (4)
C9—C8	1.343 (3)	C15—H15A	0.98 (5)
			
C9—N4—C3	133.30 (15)	N1—C2—C3	111.79 (17)
C9—N4—C5	119.96 (16)	N1—C2—C15	120.70 (18)
C3—N4—C5	106.74 (14)	C3—C2—C15	127.5 (2)
C5—N1—C2	105.47 (14)	N12—C13—C14	107.10 (18)
C7—N6—C5	116.74 (17)	N12—C13—H13A	126.4
C2—C3—N4	104.82 (16)	C14—C13—H13A	126.4
C2—C3—C10	132.26 (18)	C9—C8—C7	119.0 (2)
N4—C3—C10	122.92 (15)	C9—C8—H8A	120.5
C13—N12—N11	112.94 (17)	C7—C8—H8A	120.5
C13—N12—H30A	125.4 (15)	C13—C14—C10	105.02 (19)
N11—N12—H30A	121.7 (15)	C13—C14—H14A	127.5
N1—C5—N6	126.85 (16)	C10—C14—H14A	127.5
N1—C5—N4	111.16 (16)	N6—C7—C8	123.9 (2)
N6—C5—N4	121.98 (17)	N6—C7—H7A	118.0
C8—C9—N4	118.35 (17)	C8—C7—H7A	118.0
C8—C9—H9A	120.8	C2—C15—H15C	114 (2)
N4—C9—H9A	120.8	C2—C15—H15B	111 (2)
N11—C10—C14	110.84 (17)	H15C—C15—H15B	106 (3)
N11—C10—C3	120.54 (17)	C2—C15—H15A	110 (3)
C14—C10—C3	128.62 (17)	H15C—C15—H15A	108 (3)
C10—N11—N12	104.10 (16)	H15B—C15—H15A	107 (3)
			
C9—N4—C3—C2	−178.99 (18)	N4—C3—C10—C14	152.1 (2)
C5—N4—C3—C2	1.06 (19)	C14—C10—N11—N12	0.0 (2)
C9—N4—C3—C10	1.0 (3)	C3—C10—N11—N12	−179.70 (16)
C5—N4—C3—C10	−178.91 (16)	C13—N12—N11—C10	0.0 (2)
C2—N1—C5—N6	−178.49 (18)	C5—N1—C2—C3	0.3 (2)
C2—N1—C5—N4	0.4 (2)	C5—N1—C2—C15	−177.7 (2)
C7—N6—C5—N1	−179.55 (19)	N4—C3—C2—N1	−0.9 (2)
C7—N6—C5—N4	1.7 (3)	C10—C3—C2—N1	179.06 (18)
C9—N4—C5—N1	179.12 (16)	N4—C3—C2—C15	177.0 (2)
C3—N4—C5—N1	−0.9 (2)	C10—C3—C2—C15	−3.0 (4)
C9—N4—C5—N6	−2.0 (3)	N11—N12—C13—C14	−0.1 (3)
C3—N4—C5—N6	177.99 (17)	N4—C9—C8—C7	0.8 (3)
C3—N4—C9—C8	−179.3 (2)	N12—C13—C14—C10	0.0 (3)
C5—N4—C9—C8	0.6 (3)	N11—C10—C14—C13	0.0 (2)
C2—C3—C10—N11	151.8 (2)	C3—C10—C14—C13	179.63 (19)
N4—C3—C10—N11	−28.2 (3)	C5—N6—C7—C8	−0.2 (3)
C2—C3—C10—C14	−27.8 (3)	C9—C8—C7—N6	−1.1 (4)

### Hydrogen-bond geometry (Å, º)

**Table:**

*D*—H···*A*	*D*—H	H···*A*	*D*···*A*	*D*—H···*A*
N12—H30*A*···N6^i^	0.89 (2)	2.08 (2)	2.966 (2)	175 (2)

Symmetry code: (i) *x*−1, *y*, *z*.

## References

[bb1] An, H.-Y., Xi, B., Abassi, Y., Wang, X.-B. & Xu, X. (2009). WO Patent No. 2009023402.

[bb2] Anaflous, A., Benchat, N.-E., Ben-Hadda, T., El Bali, B. & Bolte, M. (2004). *Acta Cryst.* E**60**, o1131–o1132.

[bb3] Bruker (2000). *SMART*, *SAINT* and *SADABS* Bruker AXS Inc., Madison, Wisconsin, USA.

[bb4] Kim, J.-H., Hong, S.-H. & Hong, S.-W. (2011). *Bioorg. Med. Chem. Lett.* **21**, 6977–6981. 10.1016/j.bmcl.2011.09.118 22030027

[bb5] Linton, A., Kang, P., Ornelas, M., Kephart, S., Hu, Q.-Y., Pairish, M., Jiang, Y. & Guo, C.-X. (2011). *J. Med. Chem.* **54**, 7705–7712. 10.1021/jm2010942 21955208

[bb6] Sheldrick, G. M. (2008). *Acta Cryst.* A**64**, 112–122. 10.1107/S0108767307043930 18156677

[bb7] Yang, F.-L., Li, G.-C. & Yao, C.-S. (2008). *Acta Cryst.* E**64**, o2469. 10.1107/S160053680803941X PMC2960121 21581436

